# Mechanisms and biomarkers of immune-related adverse events in gastric cancer

**DOI:** 10.1186/s40001-023-01365-3

**Published:** 2023-11-08

**Authors:** Ping’an Ding, Pengpeng Liu, Lingjiao Meng, Qun Zhao

**Affiliations:** 1 The Third Department of Surgery, the Fourth Hospital of Hebei Medical University, Shijiazhuang, 050011 Hebei China; 2 Hebei Key Laboratory of Precision Diagnosis and Comprehensive Treatment of Gastric Cancer, Shijiazhuang, 050011 China; 3 Research Center of the Fourth Hospital of Hebei Medical University, Shijiazhuang, 050011 China

**Keywords:** Biomarkers, Gastric cancer, Immune-checkpoint inhibitors

## Abstract

Immune-checkpoint inhibitors (ICIs), different from traditional cancer treatment models, have shown unprecedented anti-tumor effects in the past decade, greatly improving the prognosis of many malignant tumors in clinical practice. At present, the most widely used ICIs in clinical immunotherapy for a variety of solid tumors are monoclonal antibodies against cytotoxic T lymphocyte antigen-4 (CTLA-4), programmed cell death protein 1 (PD-1) and their ligand PD-L1. However, tumor patients may induce immune-related adverse events (irAEs) while performing immunotherapy, and irAE is an obstacle to the prospect of ICI treatment. IrAE is a non-specific disease caused by immune system imbalance, which can occur in many tissues and organs. For example, skin, gastrointestinal tract, endocrine system and lung. Although the exact mechanism is not completely clear, related studies have shown that irAE may develop through many ways. Such as excessive activation of autoreactive T cells, excessive release of inflammatory cytokines, elevated levels of autoantibodies, and common antigens between tumors and normal tissues. Considering that the occurrence of severe IrAE not only causes irreversible damage to the patient’s body, but also terminates immunotherapy due to immune intolerance. Therefore, accurate identification and screening of sensitive markers of irAE are the main beneficiaries of ICI treatment. Additionally, irAEs usually require specific management, the most common of which are steroids and immunomodulatory therapies. This review aims to summarize the current biomarkers for predicting irAE in gastric cancer and their possible mechanisms.

## Introduction

**Fig. 1:**
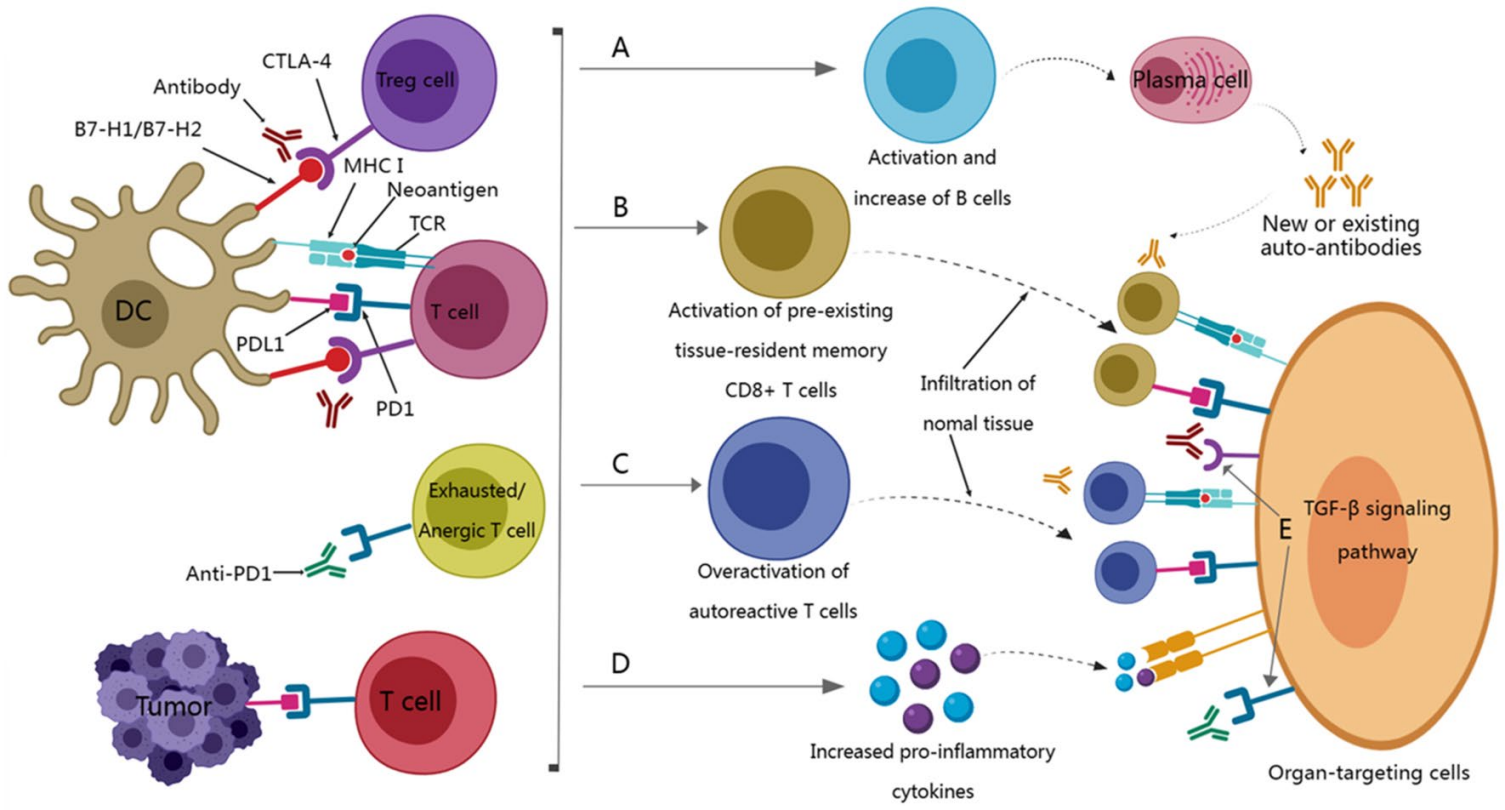
The potential mechanism of irAE related to ICI treatment. **A** Blocking CTLA-4 can induce the activation of autoreactive T cells by inducing Treg depletion and functional defects, thereby stimulating B cells to increase the production of autoantibodies. **B** Blocking CTLA4 and/or PD1 may be related to the expansion and activation of pre-existing tissue-resident memory T cells. **C** Blocking PD-1 can induce the reactivation of depleted/disabled T cells, which leads to the overactivation of autoreactive T cells. Epitope diffusion can lead to the destruction of tolerance. **D** The death of tumor cells killed by T cells can induce an increase in the level of pro-inflammatory cytokines, which in turn leads to damage to normal tissues or organs. **E** ICl may directly damage tissues or organs by binding to CTLA-4 and/or PD1 expressed in normal tissues. DC, dendritic cells; MHC, major histocompatibility complex; Treg, regulatory T cells; TCR, T cell receptor

In recent years, immune checkpoint inhibitors (ICIs) have achieved satisfactory results in various tumor types, greatly changing the treatment strategy of tumors and bringing more benefits to patients’ survival [1–3]. However, the survival benefit of cancer patients depends not only on the efficacy of ICI, but also on the occurrence of related adverse events caused by ICI, namely immune-related adverse events (irAE) [4, 5]. IrAEs are very common in ICI treatment and can occur in multiple tissues and organs [6, 7]. Among them, the most commonly affected organs for grade 3 or above irAE are the digestive system, endocrine system, and lungs, while others include the nervous system, kidneys, liver, and heart [8, 9]. Kawazoe et al. [10] found in a study evaluating the safety and efficacy of pembrolizumab combined with S-1 plus oxaliplatin as first-line treatment for advanced gastric/gastroesophageal junction cancer that the incidence of grade 3 or above irAE was 57.4%. The more common irAEs were thrombocytopenia (14.8%), neutropenia (13.0%), colitis (5.6%), and adrenal insufficiency (5.6%). Other irAEs included pneumonia, type 1 diabetes, and peripheral neuropathy. For low-grade irAEs that occur during ICI treatment, corresponding routine treatments (such as steroid hormones and immunosuppressants) can be given to relieve clinical symptoms, while high-grade irAEs may consider suspending or terminating immunotherapy [11, 12]. Previous hypotheses suggested that the mechanisms behind irAEs include overactivation of the immune system, excessive release of inflammatory cytokines, elevated levels of pre-existing autoantibodies, and the presence of shared antigens between tumors and normal tissues [9, 13, 14] (Fig. 1). However, given the relatively hidden clinical features of irAE, the immature mechanism of occurrence and lack of identification of sensitive markers that may occur irAE, which makes early prediction of patients susceptible to irAE particularly difficult [9]. Thus, understanding irAE and developing predictive biomarkers for their occurrence are crucial for achieving the maximum benefit–risk ratio for ICI-treated patients. Considering the unique clinical value, convenience and accuracy of biomarkers, we list the currently known predictive biomarkers for gastric cancer irAE in this review, providing targeted insights into irAEs as a reference for future studies.

## Potential biomarkers associated with therapeutic response to ICIs in GC

Currently, many biomarkers are used in clinical practice, including PD-L1 clinical prediction score (CPS), microsatellite instability (MSI) status, and other recognized markers such as tumor mutation burden (TMB) and gene expression score (GEP) [15–18]. However, although the above markers have certain predictive value for ICIs, their predictive ability is still not ideal, and there are contradictions in some studies. Therefore, finding reliable biomarkers is crucial for the initial identification of patients with tumors that may benefit from ICIs treatment.

Studies have shown that tissue-resident memory T cells (TRM) and tertiary lymphoid structures (TLS) are associated with good prognosis [19, 20]. Mori et al. [19] discovered through immunohistochemical detection CD103 + T cells and evaluated the relationship between CD103 + T cells and TLS that CD103 + T cells were located around TLSs, and patients with high CD103 showed rich TLS, and patients with high CD103 cells and rich TLSs had better prognosis. In addition, CD103 + CD8 + cells in GC showed better prognosis and were associated with TLS. For patients receiving ICI treatment, high CD103 and TLSs enrichment showed excellent anti-tumor immune response. Moreover, CD103 + CD8 + T cells in GC express higher levels of PD-1, granzyme B, and interferon-γ than CD103—CD8 + T cells. In a study on the distribution and proportion of TLSs and TRMs in lung adenocarcinoma, Zhao et al. [20] showed that the proportion of TRMs within TLSs was significantly higher than that outside TLSs, and was positively correlated with patient survival. In addition, CD103 + TRMs were closely related to TLS maturity, and higher maturity TLSs showed higher proportions of CD4 + CD103 + TRMs and CD8 + CD103 + TRMs. Subsequently, Nose et al. [21] also showed that in patients with ICI treatment, those with high levels of CD103 in peripheral blood CD8 + T cells had significantly higher progression-free survival than the low-level group, and the CD103 + CD8 + T cell population was mainly composed of central memory T cells, showing high Ki-67 expression and a small amount of cytotoxic particles.

Additionally, based on the whole and single-cell RNA-seq data of tumor-infiltrating immune cells, Yang et al. [22] found that tumor-infiltrating PD-1^hi^CD8 + T cells could serve as effective biomarker for ICI treatment response in multiple cancers, including GC. In clinical samples and animal models, they found that high-score PD-1^hi^CD8 + T cell subsets have better therapeutic response and longer survival time. Moreover, tumor-infiltrating PD-1^hi^CD8 + T cells showed better predictive performance when combined with tumor mutation burden (TMB), which can be used as an effective supplementary biomarker for TMB. Zhang et al. [23] established an EV-score based on four plasma EV proteins (ARG1/CD3/PD-L1/PD-L2) to predict immunotherapy results and dynamically monitor disease progression. They believe that high EV-score reflects stronger anti-tumor immune microenvironment characteristics, manifested as more activated CD8 + T/NK cells, higher Th1/Th2 ratio and higher IFN-γ/perforin/granzyme expression in peripheral blood. Among GC patients, those with an EV-score ≥ 1 can benefit more from ICIs, while those with EV-score < 1 could potentially benefit more from ICIs in combination with HER2 treatment.

According to reports, the TGFβ signaling plays a key role in cancer progression by forming the tumor structures and inhibiting the anti-tumor activity of immune cells [24]. Increasing evidence suggests that TGF-β is involved in regulating the composition and behavior of immune components in the tumor microenvironment (TME), thereby inducing tumor immune escape, especially ICI [25–27]. Related studies have confirmed that overactive TGF-β signaling is associated with ICI resistance, and the synergistic effect of TGF-β blockers and ICI can significantly reduce tumor immune tolerance [28–30]. For example, TGF-β/PD-L1 bispecific antibodies such as YM101, BiTP, and M7824 have shown strong anti-tumor activity in preclinical and clinical models. All of them showed high binding affinity to TGF-β/PD-L1 dual targets, and had better anti-tumor activity than single anti-PD-L1 or anti-TGF-β treatment [29–31]. Mechanistically, YM101 exerts anti-tumor effects by increasing the number of tumor-infiltrating lymphocytes and dendritic cells, increasing the proportion of M1/M2, and enhancing the production of cytokines in T cells [29]. BiTP demonstrates potent anti-tumor effects by reducing collagen deposition, enhancing CD8 + T cell penetration in the tumor microenvironment and increasing tumor T lymphocyte infiltration [30]. M7824 can activate dual anti-immunosuppressive functions through TME, induce anti-tumor activity by the innate and adaptive immune system, and block TGF-β1-induced tumor interstitialization and PD-L1-dependent immunosuppression potential [31–33]. In addition, TGFβ2 is highly expressed and is associated with the expression of genes drive epithelial–mesenchymal transition (EMT) in GC [34]. Meanwhile, TGFβ2 was associated with high levels of multiple immune cell infiltration and cytokine expression in GC microenvironment. The expression of PD-1, PD-L1 and CTLA-4 was significantly higher in tissues with high TGFβ2 expression, and the reactivity of immune checkpoint blockade (ICB) was significantly enhanced [35].

However, there is basically no specific signaling pathway in the process of anti-tumor immunity, such as Notch signaling pathway; PI3K–AKT signaling pathway; hedgehog signaling pathway; NF-κB signaling pathway; JAK–STAT signaling pathway; Wnt/β-catenin signaling pathway, etc [17, 36–40]. Almost all biological processes of cell proliferation and transformation involve these same signaling pathways. For example, Notch signaling is associated with anti-tumor immunity/immunotherapy [41, 42]. Co-mutations of NOTCH1-3 and homologous repair genes are associated with lasting clinical benefits [43]. Recent studies by Long et al. [44] have shown that NOTCH4 mutations have better clinical benefits in patients with gastric cancer, and NOTCH4 mutations are significantly associated with enhanced immunogenicity, including TMB, co-stimulatory molecule expression and activation of antigen processing mechanisms. In addition, Wnt/catenin pathway is also involved in tumor immunotherapy [45]. Blocking the Wnt/catenin pathway can increase the sensitivity of gastric cancer cells to PD-1 antibody.

Studies show that inflammatory biomarkers have a potential prognostic effect on ICI treatment in cancer patients [46, 47]. The GIPI nomogram established by Formica et al. [48] based on NLR, CRP and ALB showed a significant prognostic value for mGOJ/GC at the metastatic gastroesophageal junction receiving ICI. Similarly, Chen et al. [49] controlled nutritional status (CONUT) score based on total lymphocyte count (TL), total cholesterol level (T-CHOL) and serum albumin (ALB), providing a useful immunological prognostic biomarkers for cancer patients. Patients with high CONUT scores were associated with shorter PFS and OS of ICI or chemotherapy. In addition, patients with high CONUT score had lower PFS and OS in the case of PD-1/PDL1 positive expression.

With the shift towards individualized and precise therapy in the current treatment modes, circulating tumor DNA (ctDNA) has become a crucial biomarker for evaluating the therapeutic effect of ICI before or after treatment of solid tumors [50–52]. Compared to traditional marker screening, ctDNA has the following unique advantages [53, 54]. First, ctDNA detection is less invasive and only requires a minimal peripheral blood sample instead of a biopsy. Secondly, ctDNA mainly consists of genomic DNA fragments released from cell apoptosis, necrosis or active secretion. Thus, it reflects more comprehensive tumor information compared to tissue biopsy. Besides, tissue biopsy can only be further detected after tumor progression, while ctDNA can be monitored in real time. Kim et al. [55] evaluated serum ctDNA levels in ICI-treated metastatic GC and showed that decreased ctDNA was associated with improved prognosis.

Recently, research on ICI-induced irAE has also found a correlation between the occurrence of irAE and good treatment outcomes [56, 57]. Two hypotheses may explain this phenomenon [18, 58, 59]. One is that the immune response triggered in unrelated areas to the tumor may be non-specific immune or inflammatory, and the other may be the response to antigens that cross-react with tumor-associated antigens. Therefore, irAEs are considered potential clinical biomarker for predicting ICI response. Known gastric cancer ICIs related biomarkers are shown in Table 1.

**Table 1:** Potential biomarkers related to the therapeutic effect of ICIs in GC

Biomarker	Author	Year	Patient number	Correlation between biomarker and ICIs in GC
IRGPI	Zhang [60]	2022	–	IRGPI can predict the prognosis of GC patients and their response to immunotherapy, and patients with lower IRGPI may benefit more from ICI therapy
PDPN	Hu [61]	2020	65	PDPN is significantly associated with M2-type TAM and immune markers of T cell exhaustion, and high PDPN predicts poor survival outcomes, especially in GC patients with Her-2 +
ANO9	Katsurahara [62]	2021	84	High ANO9 expression is an independent poor prognostic factor in patients with advanced GC, and its depletion reduces the ability to bind to PD-1 by downregulating PD-L2 expression
CSMD1	Huang [63]	2021	557	CSMD1-mut is associated with increased TMB and favorable survival, and may have potential significance in predicting the efficacy of anti-PD-L1
mTOR	Cheng [64]	2022	1661	Mutations in mTOR pathway-related genes are associated with better survival in patients treated with ICI, and are associated with increased expression of TMB and PD1/PD-L1. Including FGFR2, PIK3C3, FGFR4, FGFR1, FGF3, AKT1, mTOR and RPTOR
MUC16	Zhang [65]	2022	1139	MUC16 mutation is associated with better prognosis, including lower LNM rate and higher OS rate. Furthermore, MUC16 mutation status was associated with TMB, microsatellite status
NOTCH3	Cui [39]	2021	48	High NOTCH3 expression was associated with lower CD8 + T cells and higher immunosuppressive cells, and NOTCH3 expression was negatively correlated with TMB, GEP score and IPRES
OX40 and LAG3	Ohmura [66]	2020	30	OX40 and LAG3 are associated with better prognosis in patients with advanced gastric cancer treated with anti-PD-1 therapy
PRKDC	Tan [67]	2020	34	PRKDC mutations are significantly associated with TMB in a variety of cancers, and gastric or colon cancer patients with PRKDC mutations are also highly associated with MSI-H
TP53	Li [68]	2020	3380	The anti-tumor immunity of TP53 mutation in STAD was significantly lower than that of TP53 wild type, and TP53 mutation cancer was more likely to have higher TMB and TAL
FAM score	Li [69]	2022	34	FAM score is associated with immune-related genomic biomarkers, immune cell infiltration and abnormal immune signaling pathways
EP300	Chen [70]	2021	–	EP300 mutant cancer has significantly higher TMB in a variety of cancer types, and shows a higher proportion of MIS-H and PD-L1 in colon cancer and gastric cancer. In addition, EP300 mutant cancers responded well to ICIs
RIPK2	Song [71]	2022	–	High RIPK2 expression is associated with poor prognosis in many cancers. Gene co-expression analysis showed that RIPK2 was positively correlated with the expression of immune checkpoint markers
circDLG1	Chen [72]	2021	73	CircDLG1 was significantly up-regulated in anti-PD-1-resistant GC tissues, and high circDLG1 promoted the proliferation, migration, invasion and immune escape of GC cells
68 Ga-FAPI-04	Rong [73]	2022	21	High FAP expression is closely related to poor prognosis and immunosuppressive cell infiltration. The high uptake of 68 Ga-FAPI-04 is associated with the reduced therapeutic efficacy of ICB therapy
CXCR4	Xue [74]	2021	–	High expression of CXCR4 is positively correlated with advanced stage and grade of gastric cancer, and is associated with poor prognosis
TM4SF18	Qin [75]	2022	40	TM4SF18 is up-regulated in GC tissues and cells and is an independent prognostic factor for GC. The expression level of M4SF19 is negatively correlated with most immune cell marker genes and is associated with many immune cells and immune pathways
HLA-I	Iwasaki [76]	2021	209	The degree of CD8 + cell infiltration was significantly reduced in HLA-I deficient tumor regions
CYT score	Hu [77]	2021	8	CYT score was positively correlated with the proportion of tumor-infiltrating CD8 + T cells and macrophages, and negatively correlated with the proportion of regulatory T cells. High CYT score showed good prognosis and was associated with PD-1, TMB, EB virus subtype and MSI. Patients who responded to anti-PD-1 therapy had a higher CYT score
LA	Kumagai [78]	2022	–	LA is an active checkpoint of Treg cell function in highly glycolytic TME and can upregulate PD-1 expression
HRD	Fan [79]	2020	484	HR gene mutation is associated with increased TMB, MSI, and enhanced immune activity. The overall survival rate of HR mut is significantly higher than that of HR wt in GC
IL-1R1	Zhang [80]	2022	409	High IL-1R1 expression indicates poor prognosis and poor response to ICB. IL-1R1 cultivates an immunosuppressive microenvironment characterized by upregulation of M2 macrophages and depletion of CD8 + T cells
Helicobacter pylori	Zhang [81]	2021	–	*Helicobacter pylori*-activated immune response improves the prognosis of GC patients by increasing PD-L1 expression and CD3 + T cells

As mentioned earlier, multiple studies have shown that the occurrence of irAE is associated with better clinical outcomes in ICI-treated various cancers [82, 83]. Hussaini et al. [84] conducted a study on the correlation between the incidence of irAE after using ICIs and the clinical prognosis of various solid tumors. They found that the occurrence of irAEs was positively correlated with ORR, PFS and OS, but not with tumor location and ICI type. Additionally, grade 3 or higher irAEs show a better ORR but worse OS. Similarly, Xu et al. [85] based on the correlation between irAE and ICI efficacy in patients with hepatocellular carcinoma (HCC) also showed that the occurrence of irAE was associated with clinical benefit. However, they believe that low-grade irAE is a better predictive marker for ICI treatment in HCC. Given that Hussaini et al. research was based on the evaluation of irAE and ICI treatment efficacy for multiple malignant tumors, while Xu et al. research object was only liver cancer patients, which may lead to differences in this result. Additionally, Xu et al.also found that patients with diarrhea/colitis, hyperthyroidism/hypothyroidism or rash had better prognosis [85]. Similarly, a previous meta-analysis also showed that the occurrence of endocrine, skin and gastrointestinal irAE was significantly associated with good prognosis in ICIs-treated patients, while other irAEs were not [86]. Considering the consistency of the results from multiple studies, the occurrence of irAE is significantly associated with excellent clinical prognosis. Therefore, can it be considered that the sensitive biomarkers predicting the occurrence of irAEs may also predict the therapeutic effect of ICI to some extent, and the biomarkers of the two are somewhat similar?

## Potential biomarkers associated with irAEs in GC

IrAEs are mainly caused by non-specific activation of the immune system and can manifest as specific and non-specific symptoms [9]. Previous hypotheses mainly focused on the excessive release of inflammatory cytokines, the overactivation of the immune system, the amplification of pre-existing abnormal antibodies, and the existence of shared antigens between tumors and normal tissues [13, 14]. Non-specific symptoms typically include fever, cough and fatigue, while specific symptoms usually involve adverse reactions of specific organs or tissues to ICI treatment in different tissues or organs, such as colitis, hyperthyroidism and interstitial pneumonia [87]. However, most common clinical ICB strategies are a combination of immunotherapy with chemotherapy or targeted therapy, making it difficult to determine whether adverse events are caused by immunotherapy alone [9, 87]. Therefore, it is urgent to develop easy-to-detect biomarkers to identify irAEs. Various candidate biomarkers associated with irAEs have been reported, including gene expression profiles, C-reactive protein, human leukocyte antigen (HLA) and gut microbiome, which can predict irAE [88–93]. However, current irAE biomarkers for gastric cancer are relatively few and lack a comprehensive summary. Known irAE biomarkers are shown in Table 2.

**Table 2:** Potential biomarkers related to irAEs in GC

Biomarker	Author	Year	Patient number	Correlation between biomarker and irAEs in GC
EV-ICOS and EV-IDO1	Jiang [95]	2022	102	GC patients with higher EVICOS or EV-IDO1 carry a lower risk of irAE with shorter intervals, representing a group of patients with better tolerance to ICI. However, they were not associated with the efficacy of immunotherapy
HLA-DR15	Yano [98]	2020	11	HLA-DR15 is significantly higher in patients with pituitary irAE than in healthy controls, which may be a predisposing factor for pituitary irAE. The mechanism may be that HLA-DR15 mediated IL-17
TLS	Mori [19]	2021	19	Patients with high TLS showed excellent anti-tumor immune response and higher frequency of immune-related adverse events
P-CRP and CA19-9	Matsunaga [118]	2022	78	CA19-9 and P-CRP are effective predictors of irAE, and the predictive ability of the combination of P-CRP and CA19-9 is much higher than that of independent P-CRP or CA19-9
NLR and PLR	Takada [123]	2022	73	Pretreatment NLR < 4.3 was significantly associated with a decreased risk of grade 3–4 irAE, and NLR change rate over 120% after treatment was significantly associated with increased risk of irAE
ALB and type I hypersensitivity	Shimozaki [127]	2021	247	The occurrence of type I hypersensitivity is associated with allergen-specific CD4 + T cells. When PD-1 is blocked by ICB, allergen-specific CD4 + T cells are activated to produce cytokines such as IFN-γ, TNF-α, and IL-5, leading to irAE

### EV-ICOS and EV-IDO1

Extracellular vesicles (EVs), nanoparticles (40–160 nm in diameter) containing various bioactive molecules, including proteins, lipids and nucleic acids, and are produced by almost all types of cells. EVs not only mediate signal transduction in cell communication, but also participate in physiological processes such as immune regulation and cancer progression [94]. Based on microarray screening and further validation, Jiang et al. [95] found that inducible T cell co-stimulatory factor (EV-ICOS) and indoleamine 2,3-dioxygenase 1 (EV-IDO1) can effectively predict and monitor irAE in ICI- treated GC and serve as biomarkers of irAE. Both the discovery and validation cohorts showed that the expression of EV-ICOS and EV-IDO1 in irAE patients was significantly lower than that those in non-IRAE patients after ICI treatment for a period of time. Furthermore, patients with high EV-ICOS and EV-IDO1 expression had longer time intervals from the start of treatment to irAE than those with low EV-ICOS and EV-IDO1 expression. Besides, EV-ICOS and EV-IDO1 levels were also positively correlated with CA72-4 levels. Taken together, these results suggest that GC patients with higher EVICOS or EV-IDO1 carry a lower risk of irAE with shorter intervals, representing a group of patients with better tolerance to ICI. However, EV-ICOS and EV-IDO1 were not associated with the efficacy of immunotherapy. Subsequently, they analyzed the tumor immune microenvironment (TIME) associated with EV-ICOS and EV-IDO1, and found that high expression of EV-ICOS and EV-IDO1 always showed higher immune cell infiltration and showed unique TIME.

### HLA-DR15

ICI-induced secondary adrenal insufficiency is considered to be a ‘pituitary viral AE’ [96, 97]. Yano et al. [98] found that DR15, B52 and Cw12 were significantly higher in the study group than in the healthy control group by comparing the frequency of HLA alleles in pituitary irAE patients and healthy control group, which may be predisposing factor for pituitary irAE. Previous studies have shown that HLA-DR15 can participate in autoimmune diseases including ulcerative colitis, multiple sclerosis and Goodpaste’s syndrome through interleukin 17 (a cytokine produced by T helper cell 17) [99–101]. Previous studies have also shown that the combination of anti-PD-1 and anti-CTLA4 antibodies increases the risk of pituitary irAE [102]. Moreover, anti-PD-1 and anti-CTLA4 inhibitors up-regulated Th1 and Th17 pathways [103, 104]. This may suggest that HLA-DR15 can be used as a predictive marker of pituitary irAE.

### Tertiary lymphatic structure (TLS)

Previous studies shown that TLSs are associated with anti-tumor immune responses and good prognosis in several cancers [105, 106]. TLS is an aggregation of various immune cells around B cells. It is similar to secondary lymphoid organs in structure and function, and plays a role in antigen preservation and activation of T cells [107, 108]. Mori et al. [19] found that high TLS showed excellent anti-tumor immune response and higher frequency of irAEs in GC patients with partial response (PR) to ICI. They believe that high TLS have stronger immune response and higher T cell activation, result in more irAE and better nivolumab efficacy. Previous have also reported that CD103 + T cells are memory T cells resident in tissues of interest as targets for immunotherapy, produce CXCL13, and are critical for the formation of TLS [107, 109]. Furthermore, various studies have shown that CD8 + T cells expressing CD103 are associated with good prognosis in some cancers, including GC, and about 70% of CD8 + TILs are resident memory T cells in GC [19, 110, 111].

### P-CRP and CA19-9

The theory that high C-reactive protein (CRP) levels are closely related to poor prognosis is well known [112, 113]. It has been reported that P-CRP (another platelet-associated inflammatory marker) is a useful prognostic indicator for various cancers, including GC [112–115]. Inflammatory cytokines, including interleukin-6 (IL-6), are mediators of tumor-associated inflammation that can lead to elevated CRP [116, 117]. Given that serum CA19-9 often increase in cancer patients, it is also used as a tumor marker for GC patients. Matsunaga et al. [118] found that CA19-9 and P-CRP were effective predictors of irAE, and the predictive ability of the combination of P-CRP and CA19-9 was much higher than that of P-CRP or CA19-9 alone.

### NLR and PLR

Neutrophil-to-lymphocyte (NLR) and platelet-to-lymphocyte (PLR) ratios, as conventional inflammatory markers, often represent poor prognosis [119, 120]. Tumor microenvironment is composed of inflammatory cells, which play an important role in tumorigenesis and tumor growth [121]. Pavan et al. reported that NLR and PLR are also important predictors for the development of irAE in advanced non-small cell lung cancer [122]. Subsequently, Takada et al. [123] evaluated NLR and PLR as early predictive markers of irAE in GC and found that 120% of NLR before treatment was significantly correlated with increased risk, while PLR did not show significant correlation. In addition, their results suggested that time-dependent changes in NLR elevation can be useful markers for predicting the development of severe irAE after immunotherapy. However, Fan et al. [124] found that PLR < 135 was associated with a higher incidence of irAE.

### ALB and type I hypersensitivity

Research shown that the incidence of irAE in patients with autoimmune diseases is higher than those without autoimmune diseases [125, 126]. Shimozaki et al. [127] found that type I hypersensitivity was associated with the occurrence of irAE and was a risk factor for irAE by analyzing the data of patients with and without irAE. Type I hypersensitivity is an allergic reaction caused by IgE binding to chemokines such as histamine, prostaglandins, and leukotrienes released by mast cells, and allergen-specific CD4 + T cells are involved in type T hypersensitivity [128]. In addition, when PD-1 is blocked by ICB, allergen-specific CD4 + T cells may be activated to produce cytokines such as IFN-γ, TNF-α, and IL-5, leading to irAE [129]. Previous studies have also reported that poor nutritional status and poor performance status (PS) according to the European Cooperative Oncology Group are associated with lower immunotherapy efficacy [130, 131]. Shimozaki et al.also found that patients with good PS showed a tendency to irAE, but did not show a significant correlation, and serum albumin level ≥ 3.6 g/dl was a risk factor for irAE [127].

## Management of irAE

**Fig. 2:**
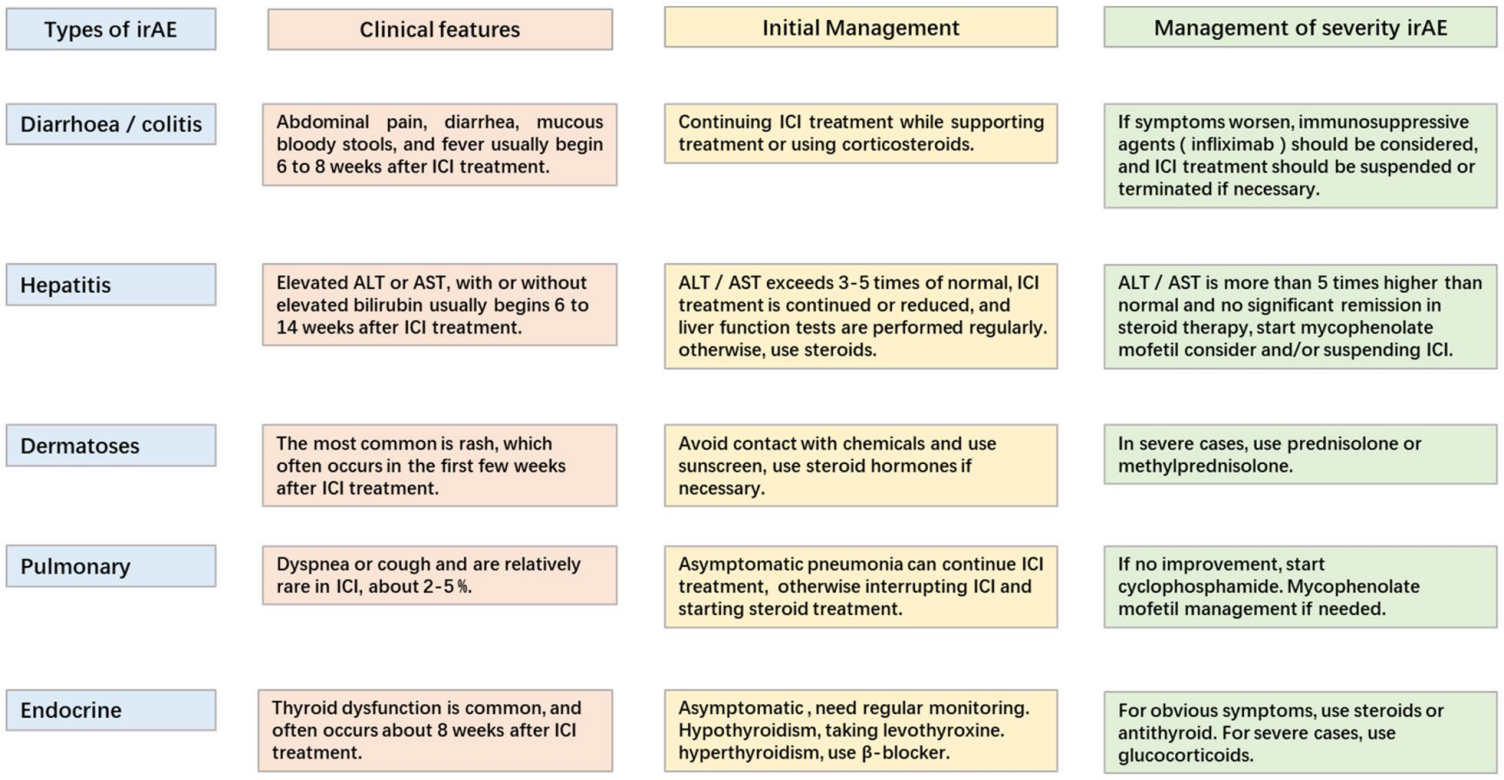
Management of immune-related adverse reactions (irAE)

Although the clinical application of ICIs against CTLA-4 and PD-1 has shown significant anti-tumor response and improved the treatment of various cancers, irAE often occurs [4, 5]. IrAE is considered to be impaired self-tolerance caused by loss of T cell suppression, involving all organ systems. Among them, skin, gastrointestinal tract, liver, endocrine system and lungs being the most common [8, 9]. The main treatment for irAE is corticosteroids or other immunosuppressive agents such as infliximab, and most irAE can be controlled through appropriate management, but may be fatal in some cases [132]. Additionally, the clinical features of irAE are relatively hidden, and the imaging findings are not obvious [9]. So early diagnosis and management of irAE is a challenge for doctors (Fig. 2).

### Gastrointestinal adverse events: diarrhea/colitis

Diarrhea or colitis is mainly manifested as abdominal pain, diarrhea, mucous bloody stools and fever, and usually begins 6–8 weeks after ICIs treatment [11]. Studies shown that the incidence of gastrointestinal irAEs is significantly higher in anti-CTLA-4 monotherapy than that of anti-PD-1/PD-L1, with the incidence of about 35%. In addition, the highest incidence of diarrhea/colitis occurs when two ICIs are combined [11, 133, 134]. NCCN guidelines [11] recommend that patients with mild diarrhea continue to receive immunotherapy and symptomatic management, while closely monitoring patients to prevent colitis symptoms. For patients with moderate diarrhea/colitis, corticosteroids are usually the preferred treatment, and over 50% of patients’ symptoms are relieved with corticosteroids. However, when corticosteroids are unable to control symptoms and diarrhea/colitis may persist or worsen, consider using infliximab (a monoclonal anti-tumor necrosis factor alpha (TNF-a)) for the treatment of various autoimmune diseases, including Crohn’s disease, ulcerative colitis, rheumatoid arthritis, etc.). For patients with grade 3 or above or severe life-threatening diarrhea/colitis, inpatient care should be considered, along with corticosteroids or immunosuppressants, and suspension or termination of immunotherapy if necessary.

### Hepatitis

Immune-related hepatitis is mainly characterized by liver dysfunction (elevated alanine transaminase (ALT) or aspartate transaminase (AST), with or without elevated bilirubin). It usually occurs 6–14 weeks after ICIs treatment. When ALT/AST reaches 3–5 times the upper limit of normal, it is recommended to continue or reduce ICI treatment, and regularly monitor liver function. If there is no improvement or deterioration, steroids should be used. When ALT/AST reaches more than 5 times the upper limit of normal and steroid treatment is not significantly relieved, consider suspending or terminating ICI [12, 135].

### Skin diseases

The most common manifestation of skin toxicity is rash, usually occurs in the first few weeks of ICI treatment, and is more common in late-stage melanoma, with an incidence of about 24.3%. When skin toxicity occurs, it is recommended that patients contact to avoid chemicals and use sun protection and other measures. In addition, for mild-to-moderate symptoms, topical use of steroids, about 4 weeks can be subsided. However, in severe cases, prednisolone or methylprednisolone can be used for treatment [136].

### Pulmonary

The incidence of pneumonia is relatively low in ICI treatment, about 2–5%. The main manifestations were dyspnea or cough. But when two ICI combinations are used, the incidence of pneumonia can reach 5–10%. ICI can be continued for asymptomatic pneumonia, but if pneumonia-related symptoms occur, consider interrupting ICI and starting steroid therapy [12].

### Endocrine

Endocrine toxicity is usually manifested as thyroid dysfunction. The onset time is usually about 8 weeks. For asymptomatic thyroid dysfunction, usually without intervention, only regular monitoring. For hypothyroidism with symptoms, it is recommended to take levothyroxine; hyperthyroidism patients recommended the use of βreceptor blocker treatment; if the symptoms are obvious, steroid or anti-thyroid medications should be considered. For severe patients, prednisolone is recommended [137, 138].

Based on the survival benefits of current ICI for a variety of advanced cancer patients and potential irAE. Ryan et al. proposed the following methods to reduce irAE in the future. First, the preventive use of drugs in high-risk populations to prevent their occurrence requires the search for reliable irAE biomarkers. Second, wait-and-see and use these immunosuppressive drugs or alter the dose and timing of ICI antibodies to maintain immune benefit while reducing immune-related toxicity. Third, alternative ICI with less toxicity and no irAE or lower irAE was developed [14].

## Conclusion

IrAE is considered to be related to the toxicity of ICI antibody mechanism. Therefore, while ensuring the therapeutic effect of ICI, the occurrence of irAE should be avoided or reduced as much as possible. To the best of our knowledge, this is the first comprehensive summary of ICI efficacy and irAE biomarkers in gastric cancer patients. Unfortunately, there are currently relatively few studies on biomarkers associated with irAE in gastric cancer, so only a small number of marker studies have been shown. Given that ICI has certain prospects for current cancer treatment, it may be accompanied by more irAE. Therefore, developing better biomarkers is critical for the management of irAE.

## Data Availability

All data and materials in our study are available upon reasonable request.
